# *Notes from the Field:* Characteristics of Million Hearts Hypertension Control Champions, 2012–2019

**DOI:** 10.15585/mmwr.mm6907a5

**Published:** 2020-02-21

**Authors:** Matthew D. Ritchey, Judy Hannan, Hilary K. Wall, Mary G. George, Laurence S. Sperling

**Affiliations:** ^1^Division for Heart Disease and Stroke Prevention, National Center for Chronic Disease Prevention and Health Promotion, CDC; ^2^Department of Medicine, Division of Cardiology, Emory University School of Medicine, Atlanta, Georgia.

Million Hearts is a national initiative co-led by CDC and the Centers for Medicare & Medicaid Services that aims to prevent 1 million heart attacks, strokes, and other related acute cardiovascular events by 2022 (*1*,*2*). On November 19, 2019, the initiative recognized 17 Million Hearts Hypertension Control Champions for achieving ≥80% blood pressure control rates among their patients with hypertension. These Champions include clinicians, practices, health centers, and health systems from 15 states that provide care for 201,045 adult patients, approximately one third (68,019) of whom have hypertension. The Hypertension Control Challenge is held annually to identify new Champions, with a call for applications in the spring, review and vetting in the summer, and announcement of Champions in the late fall. Since 2012, Million Hearts has recognized 118 Champions from 36 states and the District of Columbia who care for more than 15 million adult patients, including 5 million with hypertension (Table).*

Hypertension is a leading modifiable risk factor for heart disease and stroke (*1*–*3*). In light of this risk and the potential impact on preventing cardiovascular events by controlling hypertension, Million Hearts focuses on improving control of blood pressure among persons with hypertension. The Hypertension Control Challenge is an opportunity to call attention to the importance of controlling blood pressure in preventing cardiovascular disease and to create a sense of urgency around hypertension control, encouraging clinicians and health systems to share their achievements and promote their successful strategies.

To be eligible for recognition as a 2019 Champion, applicants were required to have an adult patient population of at least 500 persons and a hypertension control rate of 80% or higher among patients aged 18–85 years with diagnosed hypertension during a 12-month reporting period starting on or after January 1, 2018. Hypertension control was defined as a last blood pressure reading of <140/90 mm Hg, which aligns with current clinical performance measure specifications used by the health care sector to track progress in hypertension control.^†^ Using definitions consistent with these specifications nationally, approximately 75 million adults have hypertension, and only one half of these persons have their hypertension controlled (*4*). Applicants submitted information on their patient population size, demographic characteristics, and hypertension prevalence and control rates. Applicants’ eligibility status was assessed, and a subset of their submitted hypertension control data was validated through a formal external review.^§^

All 17 Champions identified in 2019 were from the private sector, including nine (53%) who had a rural-only service area and five health centers funded by the Health Resources and Services Administration. Among the 17 Champions, the median adult patient population size was 2,639 (range = 574–137,415) (Table). The median hypertension prevalence was 34% (range = 21%–52%), and the median hypertension control rate was 84% (range = 80%–98%).

**TABLE:** Characteristics of patient populations treated by Million Hearts Hypertension Control Champions, 2012–2019

Characteristic	2019	2012–2018*	Total
**Champions (total no.)**	17	101	118
**States represented, no.**	15^†^	35	37^§^
**Sector, no.**			
Private	17	98	115
Federal	0	3	3
**Service area type, no. (%)**			
Urban	7 (41)	50 (50)	57 (48)
Rural	9 (53)	19 (19)	28 (24)
Both urban and rural	1 (6)	32 (32)	33 (28)
**HRSA-funded health centers, no. (%)**	5 (29)	31 (31)^¶^	36 (31)^¶^
**No. of adult patients treated annually**			
Median (range)	2,639 (574–137,415)	6,682 (550–6,100,000)	5,706 (550–6,100,000)
Total	201,045	15,049,386	15,250,431
**Self-reported patient population characteristics, median percentage (no. of Champions with response; percentage range)**			
Minority**	20 (17; 0–92)	27 (98; 0–100)	25 (115; 0–100)
English as a second language	3 (17; 0–85)	3 (96; 0–95)	3 (113; 0–95)
Medicaid beneficiary	15 (17; 0–75)	18 (98; 0–85)	17 (115; 0–85)
Uninsured	5 (17; 0–21)	5 (18; 0–33)	5 (35; 0–33)
**No. of adult patients with hypertension treated annually**			
Median among Champions (range)	838 (189–45,704)	1,676 (96–2,900,000)	1,474 (96–2,900,000)
Total	68,019	5,023,114	5,091,133
**Median hypertension prevalence, % (range)**	34 (21–52)	30 (7–86)	31 (7–86)
**Median blood pressure control rate,^††^ % (range)**	84 (80–98)	80 (70–99)	81 (70–99)

**Abbreviation:** HRSA = Health Resources and Services Administration.

* Excludes 2016.

^†^ California, Connecticut, Florida, Illinois, Iowa, Kentucky, New Jersey, New York, Pennsylvania, Rhode Island, Texas, Utah, Virginia, Washington, and Wisconsin.

^§^ In addition to the states in which 2019 Champions were recognized, also includes the District of Columbia, Colorado, Georgia, Hawaii, Kansas, Louisiana, Maryland, Massachusetts, Michigan, Minnesota, Missouri, Montana, New Hampshire, New Mexico, North Dakota, Ohio, Oklahoma, Oregon, South Carolina, Tennessee, West Virginia, and Wyoming; the U.S. Department of Veterans Affairs was also recognized as a Champion in 2013 and has coverage throughout the United States.

^¶^ Information on HRSA-funded health center status was not consistently collected during 2012–2017; therefore, it might be underreported.

** Minority status of the patient population was determined by the applicant. No formal definition was recommended for use.

^††^ During 2012–2017, Champions were recognized for having blood pressure control rates of 70% or higher.

This national recognition program demonstrates that achieving high hypertension control rates is possible across a range of health care settings and among patient populations at high risk for uncontrolled hypertension (*1*–*5*). Various strategies have been reported by past Champions that supported their achievement of high control rates (*5*). Specific strategies highlighted by this year’s Champions included identifying a clinician within their organization who was dedicated to leading their hypertension management quality improvement efforts, arranging frequent office visits until blood pressure control was achieved, using hypertension treatment protocols and electronic health record–supported patient registries to guide patient treatment and follow-up, and providing clinician feedback through performance reports. In addition, Champions reported engaging patients in self-measured blood pressure monitoring to assess progress, inform decision-making, and encourage treatment adherence. A broader application of the strategies used by the Champions identified through the Hypertension Control Challenge could help to improve hypertension control rates nationally and decrease the incidence of heart disease and stroke.

## Footnotes

* https://millionhearts.hhs.gov/partners-progress/champions/list.html.

† http://www.qualityforum.org.

§ https://www.federalregister.gov/documents/2019/02/12/2019-01914/announcement-of-requirements-and-registration-for-the-2019-million-hearts-hypertension-control.
